# The association between frailty and dignity in community-dwelling older people

**DOI:** 10.1186/s12877-022-03056-w

**Published:** 2022-04-19

**Authors:** Fereshteh Moradoghli, Ali Darvishpoor Kakhki, Roghayeh Esmaeili

**Affiliations:** 1 Student Research Committee, School of Nursing and Midwifery, Shahid Beheshti University of Medical Sciences, Tehran, Iran; 2 Department of Medical and Surgical Nursing, School of Nursing and Midwifery, Shahid Beheshti University of Medical Sciences, Opposite to Rajaee Heart Hospital, Vali-Asr Avenue, Cross of Vali-Asr and Hashemi Rafsanjani Highway, 1996835119 Tehran, Iran

**Keywords:** Aging, Frailty, Dignity

## Abstract

**Background:**

The population of people aged 60 and older is rapidly increasing in developing countries such as Iran due to declining birth rates and increased life expectancy. Old age is associated with increased risk for frailty and reduced dignity. Frailty is a clinical syndrome characterized by depletion of physical reserves and multiple system disorders, reducing the individual’s ability to cope with stressful events. Dignity is an inherent characteristic of human beings and respecting dignity is an ethical principle. This study investigated the association of frailty with dignity among older people in Tehran, Iran.

**Methods:**

This correlational study was conducted on 200 individuals aged 60 years and older. Data collection relied on the Demographic Questionnaire, Frailty Index for Elders (FIFE) and the Patient Dignity Inventory (PDI). Data were analyzed with SPSS 25.

**Results:**

The mean age of the participants was 68 ± 5.05 years; 62% of the participants were at risk for frailty, and 69% had few dignity-related problems. The multiple regression results showed that frailty was significantly associated with dignity (ß = 0.571, *p* < 0.001). The association was significant across all the dimensions of dignity measured by the PDI. The highest predictors of frailty included dependency (ß = 0.584, *p* < 0.001), followed by existential distress (ß = 0.560, *p* < 0.001), symptom distress (ß = 0.400, *p* = 0.400), social support (ß = 0.391, *p* < 0.001), and peace of mind (ß = 0.338, *p* < 0.001) in dignity.

**Conclusions:**

The results show that higher levels of frailty in older people are associated with decreases in their dignity, and frailty was the leading predictor of dignity. Providers should develop programs to prevent and reduce frailty in those at risk and to enhance the dignity of the already frail.

## Background

This study sought to identify the relationship between frailty and dignity in older people.

Dignity is the inherent value given to a person by virtue of being human [1]. Conferring and maintaining dignity is concerned with how people feel, think, and behave regarding the value of humanity in themselves and others. To treat someone with dignity is to treat him or her as being of worth, with respect as a valued individual [2]. Respecting dignity is at the heart of ethical relationships and treatment [3]. According to the United Nations, dignity is one of the five ethical principles for older people along with independence, care, participation, and self-fulfillment. Therefore, the dignity of older people should be considered in all aspects of care and treatment [4]. A key step to help older people maintain their dignity is to identify factors related to its impairment. Physical and mental stress, chronic conditions [5], frequent visits to medical centers [6], inability, hospitalization and inadequate services [7] are among the factors that have always been associated with concerns related to dignity. When accorded dignity, people feel in control, expressing greater self-confidence and anticipating more decision-making power for themselves [8]. Therefore, it is necessary to increase the dignity of older people through programs such as listening to them without judging [9], empathetic behavior [10], creating a sense of worth [11], and maintaining independence [12]. Using the Patient Dignity Inventory (PDI), Borhani et al. [13] showed that dignity is at risk with increasing age. A study by Hall et al. [14] on cancer patients using the PDI showed that there was a correlation between decreased physical performance status and daily functioning with an increase in dignity-related problems. Also, the study of Woolhead et al. [15] showed that, with age, older people express increased concern about dignity.

Frailty is a clinical syndrome characterized by depletion of physical reserves and multiple system disorders, which reduce the body’s ability to cope with stress and maintain homeostasis, while increasing its resilience to stressful events and diseases [16]. The risk of frailty increases with aging, and thus older people are among the vulnerable groups in any society [17]. On the other hand, changes such as chronic illnesses [18], disability, unemployment, and reduced income and social support accelerate the development of frailty among older people [19]. Yang et al. [20] investigated people aged 65 years and more in Shanghai, China, using the Frailty Index, and showed that increased frailty and reduced satisfaction with life were correlated with aging. In Belgium, Hammami et al. [21] studied hospitalized older people using the Short Emergency Geriatric Assessment (SEGA) instrument, finding that 64% of the participants were at risk of frailty and 20% were frail. They also found that the rate of frailty increased with aging. A study by Siriwardhana et al. [22] on older people in Sri Lanka suggested that 15.2% were frail. Therefore, frailty is a common problem among older people.

Due to declining fertility rates and increased life expectancy, the global trend of aging populations is spreading to developing countries such as Iran. In 2020, the world had more than one billion people over 60 years old. That is 2.5 times more than in 1980 (382 million), and is projected to reach nearly 2.1 billion by 2050. By 2030, one in six people worldwide will be 60 years of age or older [23]. By 2050, about 80% of the world’s older population will reside in developing countries, with more than half in Asia [24].

The report of the United Nations indicates that while in 1975 the proportion of older people in Iran was 4.5%, this ratio will reach 10.5% in 2025 and 21.7% in 2050 [25]. Based on United Nations Population Division statistical indicators, Iran’s life expectancy is 77 years, up from 54 years in 1980 [26]. The fertility rate is 2 births per woman, down from 6.4 to 1980 [27].

Although old age is not synonymous with disease [28, 29], the need for health care services increases with an increase in the older population [30]. Among the problems of older people are decreased physical performance and independence [31], an increase in the prevalence of diseases [32], and greater need for care [33], all of which increase the risk of frailty [31]. Previous research has shown that frail older adults are more at risk for adverse health outcomes, including comorbidities [34], disabilities, reduced independence [35], hospitalization and nursing homes, medication overuse [36], cognitive problems and depression [20], negative self-perception of health, falls [37], and higher mortality rates [38]. However, the associations between frailty and dignity as one of the most basic human and moral needs in older people has been less studied.

Dignity damage is one of the most common problems in old age that can have various consequences such as shame, frustration, depression [39], increased physical and mental stress [40], and sometimes suicide [41]. Therefore, this study investigates the association between frailty, a common problem during old age, and dignity among older people.

## Methods

### Design and participants

A correlational study was conducted on people aged 60 years and above visiting the public parks and streets of Tehran. The minimum sample size was estimated to be 168 based on the study by Hulya Cakmur [34] with a confidence level of 95%, and power of 80%. Due to possible sample attrition, the final sample size sought was 200. The researchers randomly selected two parks from each region in Tehran by drawing lots for each of the city’s five regions (north, south, center, east and west), for a total of 10 parks selected from 30 main parks in Tehran. The researchers recruited 20 participants from each park. The inclusion criteria were 60 years of age and over, ability to speak Farsi (national language of Iran), Tehran citizenship, and lack of tragic events in the last six months (e.g., death of a spouse or a relative, acute illness).

### Data collection

Data was collected using a demographic characteristics questionnaire, the Frailty Index for Elders (FIFE) [42] and the Patient Dignity Inventory [PDI] [7].

Demographic characteristics collected included age, gender, marital status, education level, type of house, source of income, employment, hospitalizations during last year, annual physician visits, and chronic illnesses.

The FIFE is a robust and concise measure to identify frailty in older people. The instrument is conceptually sound with excellent content validity [42]. The FIFE items allow access to research participants’ information without the requirement of additional testing or questioning [42]. In India, Kshatri et al. [43] and Bhatt et al. [44] used the FIFE in their studies of the older people. FIFE consists of 10 items that include five health domains associated with frailty, specifically functional, physiological, psychological, social, and health care. Items in this index are answered yes (1) or no (0). Based on their total scores, the participants are placed in one of the following categories: No frailty (score of 0), Frailty risk (score of 1–3), and Frailty (score of 4 and greater) [42].

The PDI is one of the few available instruments for measuring dignity, developed by Chochinov in accordance with his model of dignity conserving care [7]. A study by Albers et al. [45] in 2011 supported that the PDI, in addition to being valid for use in terminally ill cancer patients, can also be useful in a general population such as older people to obtain insight about their dignity. Two native Farsi speakers, experienced in translating English texts, ensured that the concepts and meanings of the original questionnaire were retained [46].

The PDI includes 25 statements in five dimensions: Symptom Distress, Existential Distress, Dependency, Peace of Mind, and Social Support. Each response received from one point (not a problem) to five points (a major problem). The PDI’s possible range was from 25 to 125 with 25 indicating the highest level of dignity and 125 the lowest [7]. Scores ranging from 25 to 50 indicate a slight problem with dignity, and a score over 50 indicates a moderate-to-major dignity problem [47].

Content and face validity were examined for both the FIFE and PDI. To do this, the FIFE and PDI were given to 15 content area experts, five gerontologists, three medical ethicists, two managers of aged care facilities, and five geriatric nurses. They commented on the appropriateness, completeness, and wording of the items of the questionnaires. Also, the questionnaires were given to 20 older people to assess clarity, simplicity, and content relevance. Cronbach’s alpha was calculated for the FIFE and PDI to assess internal consistency. The value of Cronbach’s alpha for of the FIFE was 0.78 and 0.89 for the PDI, which were acceptable.

### Data analysis

This study describes the demographics, frailty, and dignity of participants. The results of Kolmogorov-Smirnov test showed that the data were normally distributed. ANOVA and t-tests were used to identify significant differences between demographic characteristics and dignity. Then, multiple regression analysis was used to predict the impact of frailty on dignity and its dimensions including existential distress, symptom distress, social support, dependency, and peace of mind, controlling for demographic variables including type of house, marital status, source of income, annual physician visits, hospitalizations during the previous month and previous year, and chronic illnesses. The tests were conducted at a significance level of 5%, and data analysis was done using Statistical Package for Social Sciences (SPSS) version 25.

## Results

The mean age of the participants was 68 ± 5.05 years, with other demographic characteristics shown in Table 1. Overall, 62% of the participants were in the frailty-risk group and 19% were in the frailty group. Moreover, 69% of the participants scored as low for dignity problems while 28% scored moderate to high for dignity problems.

**Table 1:** Demographic characteristics of the participants

Characteristics	*N* = 200	%
Gender		
Female	121	60
Male	79	40
Marital status		
Single	60	29
Married	139	71
Type of house		
Rental	47	23
Personal	146	77
Education level		
Illiterate	12	6
Primary	54	27
High school	76	38
University	58	29
Employment		
Unemployed and Housewife	50	25
Retired	120	60
Employed	30	15
Source of income		
Working	34	16
Retirement	146	75
No income	20	9
Annual physician visits		
None	48	24
1–3	97	48
≥ 4	55	28
Hospitalizations during last year		
None	150	75
1	34	17
≥ 2	16	8
Chronic illness		
No	89	45
Yes	104	55

Frailty, chronic illness, type of house, annual physician visits, hospitalizations during last year, marital status, and source of income were entered into the regression analysis as independent variables based on the ANOVA and t-tests results (Table 2). After controlling for demographic variables, two variables significantly predicted dignity: frailty and annual physician visits, with frailty being the strongest predictor. Further multiple regression analysis showed that two variables, frailty and chronic disease, could significantly predict the symptom distress dimension of dignity and that frailty was the best predictor of symptom distress. Frailty was the only predictor of the existential distress dimension of dignity and the dependency dimension. Frailty and hospitalizations during last year were significant predictors of the peace-of-mind dimension of dignity, with frailty as the leading predictor. Furthermore, the variables of frailty and chronic disease could significantly predict the social support dimension of dignity, and frailty was the best predictor of the social support dimension. The study also showed that with increasing frailty, the dignity of older people is further damaged. The most significant associations (**ß**) were between dependency and frailty, dignity and frailty, existential distress and frailty, symptom distress and frailty, social support and frailty, peace of mind and hospitalizations during last year, peace of mind and frailty, social support and chronic illness, symptom distress and chronic illness, and dignity and annual physician visits (Table 3).

**Table 2:** Relationship between dignity and other variables

Variable	Category	Mean	SD	F/t-value	*P*-value
Marital status	Single	46.70	16.04	2.27	0.024
	Married	41.29	14.66		
Type of house	Rental	47.23	18.88	2.27	0.028
	Personal	41.49	13.64		
Source of income	Working	39.00	13.10	7.90	<0.001
	Retirement	42.45	15.45		
	No income	56.29	14.46		
Annual physician visits	None	36.91	12.44	12.51	< 0.001
	1–3	42.52	14.53		
	≥ 4	53.38	17.70		
Hospitalizations during last year	None	40.82	14.35	10.91	< 0.001
	1	48.77	14.49		
	≥ 2	57.38	19.03		
Chronic illness	No	38.06	10.68	-4.24	< 0.001
	Yes	47.01	17.26		

*SD* Standard Deviation

In PDI, a higher score indicated a lower level of dignity

**Table 3:** The results of multiple linear regression analysis predicting dignity and its dimensions

Dependent variable	Predictive variables	SD	ß	*P*-value	Confidence	
					**Lower limit**	**Upper** **limit**
Dignity	Annual physician visits	0.400	0.153	0.048	0.006	0.303
	Frailty	0.602	0.571	< 0.001	0.427	0.714
Symptom distress	Chronic illness (yes)^a^	0.979	0.156	0.050	-0.0002	0.312
	Frailty	0.293	0.400	< 0.001	0.230	0.569
Existential distress	Frailty	0.195	0.560	< 0.001	0.409	0.710
Dependency	Frailty	0.065	0.584	< 0.001	0.426	0.740
Peace of mind	Hospitalizations during last year	0.504	0.387	0.009	0.097	0.676
	Frailty	0.138	0.338	< 0.001	0.157	0.518
Social support	Chronic illness(yes)^a^	0.605	0.187	0.014	0.038	0.335
	Frailty	0.181	0.391	< 0.001	0.230	0.437
Note: In the	Dignity *R* = 0.720	R2 = 0.518	ADJ.R2 = 0.484	*P* = < 0.001		

*ß* standardized beta

Reference group: No^a^

## Discussion

The aim of this study was to investigate the association between frailty and dignity among older people in Tehran. The results showed that 62% of older people in Iran were at risk for frailty. This finding is consistent with prior studies. Nguyen et al. [48] found that 65.6% of older hospitalized Vietnamese people were at risk of frailty. Siriwardhana et al. [22] found a 48.5% rate of frailty among older people living in Kegalle Village in Sri Lanka.

Most of the older people in the present study displayed problems with dignity consistent with a review study by Jacelon et al. [49]. Decreased dignity is a threat to one’s health associated with emotional changes, fear, despair, feelings of worthlessness, insecurity, loneliness, depression and sometimes suicide [39].

In research by Wang et al. [47] which aimed to investigate dignity and its influencing factors in patients with cancer with using the PDI, the main dignity problem was related to the dimension of symptom distress. Older people are more exposed to stress due to decreased self-esteem, loss of friends and relatives, decreased physical independence, and chronic diseases [50]. Therefore, identifying different sources of stress experienced by older people could contribute to interventions that increase dignity. In the study by Borhani et al. [13] on hospitalized patients using the PDI, the greatest concerns expressed by patients were predominantly in the dimension of symptom distress followed by the dimension of peace of mind. But in the present study, subjects expressed most concern about peace of mind after symptom distress, which among older people can be due to illness, reduced income, increased dependence, and lack of privacy.

Participants in the present study expressed fewer problems related to dependency, possibly because participants in this study were active, having been recruited in a public park. They also expressed fewer problems unlike hospitalized participants who listed dependence as a major problem [13]. Such findings suggest that encouraging activity can contribute to a feeling of independence. Participants in the present study pointed to reduced social support as one of the main dimensions of reduced dignity and cited the need for ongoing support such as pensions and health insurance.

Feng et al. [51] found that frailty predicts decreased life expectancy and increased depression in older people. Therefore, identifying frail older people and performing early interventions to prevent the progression of frailty and related adverse consequences [52] can help maintain and improve dignity.

In this study frailty predicts the dimensions of dependence, existential distress, symptom distress, social support and peace of mind of dignity in older people after controlling for demographic factors. Frailty has the greatest impact on the dependency dimension, increasing dependence. Findings by Koyama et al. [53], using the Kihon Check list (KCL) to assess frailty, showed that the condition can predict dependency in older people and frail participants, and participants without frailty are significantly more dependent after discharge from the hospital.

This study’s results showed that frailty is associated with existential and symptom distress in older people. Frail elders have higher levels of chronic illness, disability, depressive symptoms, and cognitive impairment than older people without frailty [54], and these factors can increase stress. Therefore, identifying and managing frailty can help reduce stress and adverse health consequences in older people. This study’s results showed that increasing frailty is associated with reduced social support in older people. Research by Mehrabi and Beland [54] showed that frail middle-aged people reported less participation in social activities and less social support than older people not frail. Also, older people who received less social support were more likely to suffer from depression or cognitive impairment. Therefore, programs for frail older people should include methods for improving social support. Finally, increasing frailty reduces mental peace in older people. A study by Mulasso et al. [55] on older people showed that frailty is highly associated with the aggravation of psychosocial problems and symptoms of depression, social isolation and loneliness in frail older people more so than with non-frail older people. Therefore, by reducing frailty, peace of mind in older people can be improved.

The results of this study showed that more physician visits following disease in older people led to reduced dignity. A study by Anderberg et al. [56] showed that the dignity of older people is further threatened following illness and hospitalization. Also, Baillie et al. [57] showed that seeing a physician and getting sick are the main factors threatening the dignity of older people.

Therefore, reducing the number of hospitalizations for older people through treatment and remote care, such as telemedicine and telenursing, as well as through programs for the prevention and management of chronic diseases can improve dignity. Likewise, this study suggests that increased number of hospitalizations in older people can reduce peace of mind. A review study by Levenson [58] in England suggested that multiple care techniques and manner of treatment can threaten dignity among older people. Other acts endangering dignity include lack of attention to patient appearance, nurse and patient not being the same gender, mixed wards, using inappropriate words to address the patient, non-observance of patient privacy, and inappropriate communication between caregivers and patients [59]. Therefore, to improve feelings of dignity, medical and care staff should have sufficient knowledge and skills to manage and treat older people with dignity and have sufficient knowledge about the factors that reduce the dignity and its dimensions in medical settings. Care should respect the dignity of the patient. Good nursing care aims at the enhancement of the dignity of the human person in all his or her dimensions and also succeeds in realizing this intention in practice. Hence, good nursing care is to be considered as dignity-enhancing care. To determine whether a nursing act, attitude, or instrument is morally good, one must apply the criterion of dignity of the human person, considered in all dimensions [60].

Also, the results showed that chronic illness in older populations can increase symptom distress and decrease social support in the dimensions of the dignity. Research by Amininasab et al. [61] on patients with heart failure using the PDI showed decreased dignity among older people with increased numbers of chronic diseases. This can be due to the effects of chronic illness such as feelings of being an imposition others and hopelessness [62], decreased self-efficacy [63], limited physical and mental activity and reduced quality of life [64], and the effects of multiple visits to medical centers [52]. Therefore, programs to prevent and manage chronic diseases may reduce symptom distress and increase access to social support for older people.

This study had a number of limitations. One important limitation was that the study population was not homogeneous and older participants in the study had various conditions in terms of health and disease. Also, the study population was selected on a nonprobability basis from 10 parks in Tehran and the total sample size was limited. Therefore, it is suggested that further studies be conducted with a larger sample size and in more restricted settings such as nursing homes and hospital.

## Conclusions

In summary, we examined the association between frailty and dignity among community-dwelling older adults in Tehran, Iran. This study showed that a significant proportion of older Iranians report experiencing frailty and dignity problems. The results indicate that with increasing frailty, the dignity of older people decreases. Our results showed that frailty is the leading predictor of dignity and also dimensions of dependence, existential distress, symptom distress, social support, and peace of mind. Therefore, it is possible that programs focused on increasing competence and preparedness in caring for older people, economic and social support, education on the causes and symptoms of frailty, attention to the differences in needs, monitoring the behavior and knowledge by caregivers can lead to a reduction in frailty, thus maintaining and improving overall dignity. Also, identifying reasons and decreasing the number of annual physician visits will help to improve the dignity of older people.

In addition, results of this study showed that chronic diseases and hospitalizations during the last year are associated with a decrease in dignity on dimensions of symptom distress, social support, and peace of mind in older people. Therefore, nurses and nursing managers can maintain and promote the dignity of older people by identifying and controlling modifiable risk factors of chronic diseases and reducing the number of hospitalizations in older people, developing nursing and tele-medicine systems, reducing the length of hospital stay, creating elder friendly medical centers, protecting privacy and ensuring proper caregiver communications, increasing the awareness of the medical staff about patient rights, increasing physical and financial independence and increasing the quality of life and financial independence, and paying attention to dignity during treatment.

## Data Availability

The datasets used and/or analysed during the current study are available from the corresponding author on reasonable request.

## Abbreviations

FIFE: Frailty Index for Elders
PDI: Patient Dignity Inventory
SEGA: Short Emergency Geriatric Assessment
SPSS: Statistical Package for Social Sciences

## References

[1] Van Gennip H, Isis E, Roelin W, Pasman H, Oosterveld-Vlug MG, Willems DL (2013). The development of a model of dignity in illness based on qualitative interviews with seriously ill patients. Int J Nurs Stud.

[2] Baillie L, Gallagher A, Wainwright P (2008). Defending dignity: opportunities and challenges for nursing.

[3] Jackson A, Irwin W. Dignity, humanity and equality: Principle of Nursing Practice A. Nurs Stand. 2011;25(28):35–7. 10.7748/ns2011.03.25.28.35.c8396 21488447

[4] United Nations. United Nations principles for older persons. United Nations New York; 1991.

[5] Ripamonti CL, Buonaccorso L, Maruelli A, Bandieri E, Pessi MA, Boldini S (2012). Patient dignity inventory (PDI) questionnaire: The validation study in Italian patients with solid and hematological cancers on active oncological treatments. Tumori.

[6] Avestan Z, Rahmani A, Heshmati-Nabavi F, Mogadasian S, Faghani S, Azadi A (2015). Perceptions of Iranian Cancer Patients Regarding Respecting their Dignity in Hospital Settings. Asian Pac J Cancer Prev.

[7] Chochinov HM, Hassard T, McClement S, Hack T, Kristjanson LJ, Harlos M (2008). The patient dignity inventory: a novel way of measuring dignity-related distress in palliative care. J Pain Symptom Manage.

[8] Hardy S (2013). Dignity in health care for people with learning disabilities.

[9] Randers I, Mattiasson A (2004). Autonomy and integrity: upholding older adult patients’ dignity. J Adv Nurs.

[10] Slettebo A, Caspari S, Lohne V, Aasgaard T, Naden D (2009). Dignity in the life of people with head injuries. J Adv Nurs.

[11] Woolhead G, Tadd W, Boix-Ferrer JA, Krajcik S, Schmid-Pfahler B, Spjuth B (2006). “Tu” or “Vous?”: A European qualitative study of dignity and communication with older people in health and social care settings. Patient Educ Counsel.

[12] Tabari F, Khaghanizade M, Dehghan-Nayeri N, Najafi-Mehri S (2016). Explain the concept of autonomy in the maintain dignity elderly: a qualitative study. Iran J Nurs Res.

[13] Borhani F, Abbaszadeh A, Moosavi S. Status of human dignity of adult patients admitted to hospitals of Tehran. J Med Ethics Hist Med. 2014;7(1):20–8. PMC4648216 26587200

[14] Hall S, Davies JM, Gao W, Higginson IJ (2014). Patterns of dignity-related distress at the end of life: a cross-sectional study of patients with advanced cancer and care home residents. Palliat Med.

[15] Woolhead G, Calnan M, Dieppe P, Tadd W (2004). Dignity in older age: what do older people in the United Kingdom think?. Age Ageing.

[16] Fried LP, Tangen CM, Walston J, Newman AB, Hirsch C, Gottdiener J (2001). Frailty in older adults: evidence for a phenotype. J Gerontol Biol Med Sci.

[17] Cesari M, Calvani R, Marzetti E (2017). Frailty in Older Persons. Clin Geriatr Med.

[18] Soysal P, Stubbs B, Lucato P, Luchini C, Solmi M, Peluso R (2016). Inflammation and frailty in the elderly: a systematic review and meta-analysis. Ageing Res Rev.

[19] Walsh SA (2010). Conducting research with the elderly: ethical concerns for a vulnerable population. South Online J Nurs Res.

[20] Yang F, Gu D, Mitnitski A (2016). Frailty and life satisfaction in Shanghai older adults: The roles of age and social vulnerability. Arch Gerontol Geriatr.

[21] Hammami S, Zarrouk A, Piron C, Almas I, Sakly N, Latteur V (2020). Prevalence and factors associated with frailty in hospitalized older patients. BMC Geriatr.

[22] Siriwardhana DD, Weerasinghe MC, Rait G, Scholes S, Walters KR (2020). Association between frailty and disability among rural community-dwelling older adults in Sri Lanka: a cross-sectional study. BMJ open.

[23] World Health Organization.The decade of healthy ageing. baseline report, Geneva, Licence: CC BY-NC-SA 3.0 IGO.2020.

[24] Dsouza SA, Rajashekar B, Dsouza H, Kumar K (2014). Falls in Indian older adults: a barrier to active ageing. Asian J Gerontol Geriatr.

[25] Sharifi F, Fakhrzadeh H, Varmaghani M, Arzaghi SM, Khoei MA, Farzadfar F (2016). Prevalence of dementia and associated factors among older adults in Iran: National Elderly Health Survey (NEHS). Arch Iran Med.

[26] Life expectancy at birth, total (years) - Iran, Islamic Rep. | Data https://data.worldbank.org/indicator/SP.DYN.LE00.IN?locations=IR. Accessed 23 Aug 2021.

[27] Fertility rate, total (births per woman) - Iran, Islamic Rep. | Data https://data.worldbank.org/indicator/SP.DYN.TFRT.IN?locations=IR. Accessed 23 Aug 2021.

[28] Pinheiro SB, Cardenas CJd, Akaishi L, Dutra MC, Martins WR (2016). Evaluation of balance and fear of falling in elderly individuals before and after senile cataract surgery. Rev Bras Geriatr Gerontol.

[29] Sadock B, Sadock V. Gangguan Somatoform dan Gangguan Nyeri. Kaplan & Sadock’s Concise Textbook of Clinical Psychiatry (Indonesian Translation Version). 2017:270 – 80.

[30] Barnett I, Van Sluijs EM, Ogilvie D (2012). Physical activity and transitioning to retirement: a systematic review. Am J Prev Med.

[31] Zhang Q, Guo H, Gu H, Zhao X (2018). Gender-associated factors for frailty and their impact on hospitalization and mortality among community-dwelling older adults: a cross-sectional population-based study. PeerJ.

[32] De A, Ghosh C (2017). Basics of aging theories and disease related aging-an overview. PharmaTutor.

[33] Beard JR, Bloom DE (2015). Towards a comprehensive public health response to population ageing. J Lancet.

[34] Cakmur H (2015). Frailty among elderly adults in a rural area of Turkey. Med Sci Monit.

[35] Coelho T, Paul C, Gobbens RJ, Fernandes L (2015). Determinants of frailty: the added value of assessing medication. Front Aging Neurosci.

[36] Kojima G (2015). Frailty as a predictor of future falls among community-dwelling older people: a systematic review and meta-analysis. J Am Med Dir Assoc.

[37] De Albuquerque Sousa ACP, Dias RC, Maciel ACC, Guerra RO (2012). Frailty syndrome and associated factors in community-dwelling elderly in Northeast Brazil. Arch Gerontol Geriatr.

[38] Chang SF, Lin PL (2015). Frail phenotype and mortality prediction: a systematic review and meta-analysis of prospective cohort studies. Int J Nurs Stud.

[39] Tadd W, Hillman A, Calnan S, Calnan M, Bayer T, Read S (2011). Right place–wrong person: dignity in the acute care of older people. Qual Ageing.

[40] Mansfield A, Nathanson V, Jayesinghe N, Foyle G (2011). The psychological and social needs of patients. BMJ.

[41] Uei SL, Wu S (2010). Promoting dignity in long term care. Macau J Nurs.

[42] Tocchi C, Dixon J, Naylor M, Jeon S, McCorkle R (2014). Development of a frailty measure for older adults: the frailty index for elders. J Nurs Meas.

[43] Kshatri JS, Palo SK, Bhoi T, Barik SR, Pati S (2020). Associations of multimorbidity on frailty and dependence among an elderly rural population: Findings from the AHSETS study. Mech Ageing Dev.

[44] Bhatt JV, Raval P, Patel U (2019). Do Females Are More Frail Than Man? A Cohort Study. Indian J Appl Basic Med Sci.

[45] Albers G, Pasman HRW, Rurup ML, de Vet HC, Onwuteaka-Philipsen BD (2011). Analysis of the construct of dignity and content validity of the patient dignity inventory. Health Qual Life Outcome.

[46] World Health Organization. Process of translation and adaptation of instruments. 2006. Available from: http://www.who.int/substance_abuse/research_tools/translation/en/. Accessed 6 Sept 2019.

[47] Wang L, Wei Y, Xue L, Guo Q, Liu W (2019). Dignity and its influencing factors in patients with cancer in North China: a cross-sectional study. Curr Oncol.

[48] Nguyen AT, Nguyen LH, Nguyen TX, Nguyen HTT, Nguyen TN, Pham HQ (2019). Frailty Prevalence and Association with Health-Related Quality of Life Impairment among Rural Community-Dwelling Older Adults in Vietnam. Int J Environ Res Publ Health.

[49] Jacelon CS, Dixon J, Knafl KA (2009). Development of the attributed dignity scale. J Res Gerontol Nurs.

[50] Arman M (2014). The comparison of depression, anxiety and stress between active and inactive old women in Isfahan. J Rehab Med.

[51] Feng L, Nyunt MSZ, Feng L, Yap KB, Ng TP. Frailty predicts new and persistent depressive symptoms among community-dwelling older adults: findings from Singapore longitudinal aging study. J Am Med Dir Assoc. 2014;15(1):76–7. 10.1016/j.jamda.2013.10.001 24314697

[52] Van Assen MA, Pallast E, El Fakiri F, Gobbens R (2016). Measuring frailty in Dutch community-dwelling older people: Reference values of the Tilburg Frailty Indicator (TFI). Arch Gerontol Geriatr.

[53] Koyama S, Katata H, Ishiyama D, Komatsu T, Fujimoto J, Suzuki M (2018). Preadmission frailty status as a powerful predictor of dependency after discharge among hospitalized older patients: A clinical-based prospective study. Geriatr Gerontol Int.

[54] Mehrabi F, Beland F, Health P (2021). Frailty as a Moderator of the Relationship between Social Isolation and Health Outcomes in Community-Dwelling Older Adults. Int J Environ Res Publ Health.

[55] Mulasso A, Roppolo M, Giannotta F, Rabaglietti E (2016). Associations of frailty and psychosocial factors with autonomy in daily activities: a cross-sectional study in Italian community-dwelling older adults. Clin Interv Aging..

[56] Anderberg P, Lepp M, Berglund AL, Segesten K (2007). Preserving dignity in caring for older adults: a concept analysis. J Adv Nurs.

[57] Baillie L (2009). Patient dignity in an acute hospital setting: a case study. Int J Nurs Stud.

[58] Levenson R. The Challenge of Dignity in Care: Upholding the Rights of the Individual: A Report for Help the Aged: Help the Aged; 2007.

[59] Birrell J, Thomas D, Jones CA (2006). Promoting privacy and dignity for older patients in hospital. Nurs Stand.

[60] Gastmans C (2013). Dignity-enhancing nursing care: a foundational ethical framework. Nurs Ethics.

[61] Amininasab S, Azimilolaty H, Moosazadeh M, Shafipour V (2016). Studying the Factors Threatening Human Dignity in Patients with Heart Failure Iranian Journal of Nursing Research. Iran J Nurs Midwifery Res.

[62] Wilson KG, Curran D, McPherson CJ (2005). A burden to others: a common source of distress for the terminally ill. Cognit Behav Ther.

[63] Sohrabi MB, Zolfaghari P, Mahdizade F, Aghayan S-M, Ghasemian-Aghmashhadi M, Shariati Z (2008). Evaluation and comparison of cognitive state and depression in elderly admitted in sanitarium with elderly sited in personal home. J Knowl Health.

[64] Canbaz S, Sunter A, Dabak S, Peksen Y (2003). The prevalence of chronic diseases and quality of life in elderly people in Samsun. Turk J Med Sci.

